# Coronary ectasia due to primary aldosteronism in an exceptional presentation

**DOI:** 10.21542/gcsp.2021.22

**Published:** 2021-10-30

**Authors:** Hammam Rasras, Falmata Laouan Brem, Noha El Ouafi, Nabila Ismaili

**Affiliations:** 1 Department of Cardiology, Mohammed VI University Hospital of Oujda, Mohammed First University of Oujda, Morocco; 2 Laboratory of Epidemiology, Clinical Research and Public Health, Faculty of Medicine and Pharmacy, Mohammed the First University of Oujda, Morocco

## Abstract

Primary aldosteronism as a cause of coronary ectasia has been reported only once in the literature and was associated with an aortic aneurysm. Here, we report a second presentation in our cardiology department - a 59-year-old female patient who was admitted for unstable angina. Coronary angiography revealed an ectasia of two major coronary arteries. An etiological assessment revealed an idiopathic primary aldosteronism.

## Introduction

Coronary ectasia (CE) is characterized as diffuse dilation of a coronary artery with a diameter 1.5 times that of a normal adjacent artery for more than one-third of its length, with a prevalence of 1%–5% and a 4:1 male-to-female ratio^1^. The most common cause is atherosclerosis, while primary aldosteronism (PA) is an unusual etiology. Coronary angiography remains the gold standard for diagnosis^2^. Its prognosis varies depending on the clinical and radiological manifestations, as well as the pace at which it is treated.

## Clinical presentation

**Figure 1.**
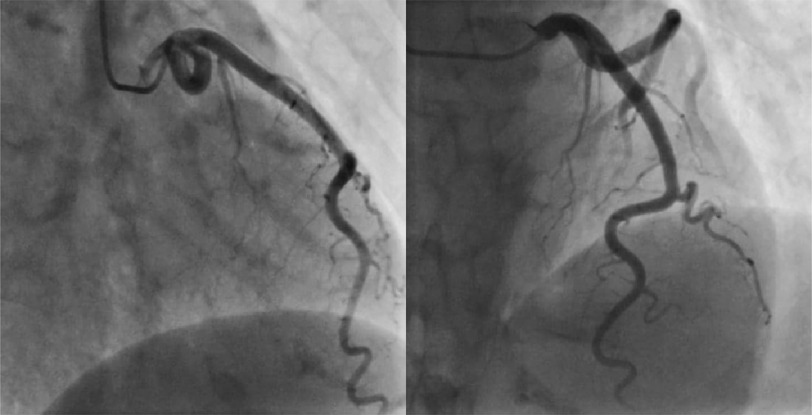
Coronary angiography revealed a left anterior descending (LAD) artery ectasia without any significant obstruction.

**Figure 2.**
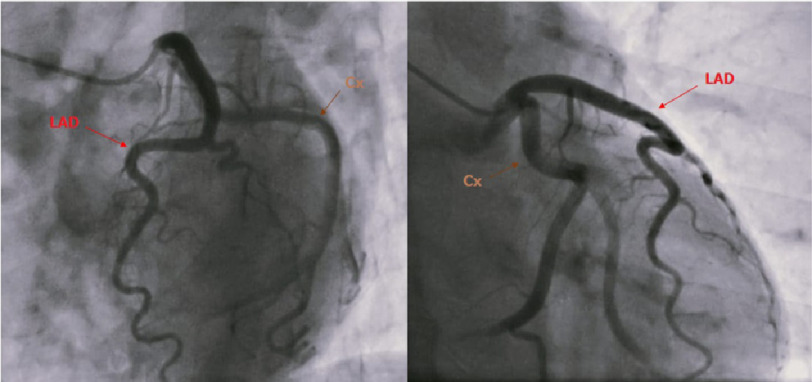
Coronary angiography revealed a circumflex coronary artery ectasia without any significant obstruction.

A 59-year-old woman, with a past medical history of treatment-resistant hypertension, presented with unstable angina with a hypertensive peak at 200/100 mmHg. Physical examination was unremarkable. Blood tests revealed hypokalemia (3 mmol/L). A transthoracic echocardiogram (TTE) showed left ventricular hypertrophy, with good LV systolic dysfunction (EF at 53%). Coronary angiography (CA) revealed ectasia of the left anterior descending (LAD) artery (Figure 1) and the circumflex coronary artery, with TIMI flow at grade 3 (Figure 2).

The patient was administered aspirin, bisoprolol and statin. A further investigation was conducted due to treatment-resistant hypertension and hypokalemia, revealing plasma aldosterone at 973 pmol/L; and plasma renin activity at 5.2 mUI/L, with a high aldosterone/renin ratio that was 98 & 91, respectively, in the lying and standing positions.

An abdominal CT scan did not show adenoma or hyperplasia of the adrenal glands. The patient was diagnosed with idiopathic PA and treated with spironolactone, leading to very good control of blood pressure. For her angina, she is still on the same therapy in addition to trimetazidine, again with good outcomes.

## Discussion

With a prevalence of 50%, atherosclerosis is the most common cause of CE. Kawasaki disease is frequent in children; approximately 17% of cases of CE are congenital, and 10–20% of them are linked to inflammatory or connective tissue disease, followed by inherited collagen defects, and iatrogenic causes such as percutaneous intervention (PCI) and PA, which are quite rare^1^.

Arshad et al. identified CE caused by PA for the first time in 1999 and associated it with aortic aneurysm^3^.

Its pathophysiology is controversially proposed as endothelial dysfunction, chronic inflammation, and fibrosis, induced by a high level of aldosterone in the blood, which can then cause an exaggerated expansive remodeling of the media, which may be responsible for media thinning^4^. Therefore, the treatment is mainly etiological, either by surgical eradication or medical therapy with anti-aldosterone, and depending on the case can sometimes be combined with corticosteroids to fight chronic inflammation^5–7^.

The ectatic segment causes a slow flow intra-coronary that promotes the development of thrombi, as well as a high risk of vasospasm. Here, antiplatelet and anticoagulation are indicated for avoiding thrombus formation and improving the flow. Trimetazidine also helps improve coronary flow by multiplying adenosine levels^7,8^. Care is required with nitroglycerin and nitrate derivatives, which have a negative impact since they induce coronary dilatation, worsening the situation and possibly causing unstable angina.^7^

For the diagnosis and evaluation of CE, coronary angiography (CA) not only remains the “gold standard”, but it also plays an important role in the description of lesions by describing their size, position, the presence or absence of stenosis or thrombus, and the degree of stenosis or thrombus if it occurs^2^.

The aim of treatment is to avoid myocardial ischemia and reduce the risk of thrombosis caused by inflammation and slow flow. Medical treatment is based on the introduction of the agents mentioned above. Antiplatelet agents and anticoagulants are essential (depending on intra-coronary flow), and statins often have their place in the presence of atheroma^5^.

In cases of thrombi or related stenosis, primary PCI with thrombus aspiration is recommended. PCI with polytetrafluoroethylene (PTFE)-covered stents can also be suggested if angina continues after maximal medical therapy. It has recently gained popularity owing to its ability to prevent coronary dilation^9^. Surgical revascularization of the ectatic segment by ligating the proximal and distal segments and replacing them with a bypass graft is rarely recommended, particularly in patients with recurrent complications^2^.

## What have we learned?

CE is rare^10^ and although its etiologies are numerous, atherosclerosis seems the most common. It is exceptional to report PA as a cause of CE. The treatment is medical, interventional or surgical, depending on the clinical and radiological situations. What is important is not to treat what is superficial, but to look for an etiology, because well-targeted management will provide means and techniques that are capable of improving the prognosis of our patients.

## Conflicts of interest

All authors report no conflicts of interest.

### Financial support

Authors declare that there was no financial support for this article.

### Author contributions

Hammam Rasras: CONCEPTION, LITERATURE REVIEW, ANALYSIS, DATA COLLECTION, WRITING, REVIEW & EDITING.

Falmata Laouan Brem: CONCEPTION, ANALYSIS, DATA COLLECTION, WRITING, REVIEW & EDITING.

N. El Ouafi: CONCEPTION, METHODOLOGY, SUPERVISION.

N. Ismaili: CONCEPTION, METHODOLOGY, SUPERVISION.
